# ‘We just been forced to do it’: exploring victimization and agency among internally displaced young mothers in Bogotá

**DOI:** 10.1186/s13031-019-0205-1

**Published:** 2019-06-03

**Authors:** Yazmin Cadena-Camargo, Anja Krumeich, Maria Claudia Duque-Páramo, Klasien Horstman

**Affiliations:** 1 Research school CAPHRI, Department of Health, Ethics, and Society, Faculty of Health, Medicine, and Life Sciences, Maastricht University, P.O. Box 616, 6200 MD Maastricht, The Netherlands; 2 Faculty of Medicine, Department of Preventive and Social Medicine, Pontificia Universidad Javeriana, Bogotá, Colombia; 3 Independent Consultant, Bogotá, Colombia

**Keywords:** Internally displaced women, Victims, Agency, Survivors, Colombia

## Abstract

**Background:**

Armed conflict in Colombia has a history of 50 years that continues to this day. According to the Victims Record of Colombia, from 1985 to 2013 2.683.335 women have been victims of the armed conflict. Women have been described as the main victims of the armed conflict, especially in the Colombian cultural context that in some regions is still considered to be a ‘machista’ and patriarchal one. In contrast, some authors have explicitly stressed Colombian women’s agency instead of positioning them only as victims. Some of them are described as ‘survivors’ of the war, emphasizing their impressive resistance to the outcomes of war and forced displacement. In contrast to the background of these scholarly discussions, our study focused on how displaced women living in Bogotá themselves articulate their experiences of agency and victimization. This paper will therefore explore how women, in reconstructing their life stories, expressed the tussles between victimization and agency.

**Methods:**

We used qualitative methods conducted within an ethnographic approach. Based on ten years of experience in the neighborhood and one year of fieldwork, we collected the life stories of twenty internally displaced mothers, and ran eight workshops with them. We analyzed the narratives with a specific focus on how women expressed victimization and agency in four important periods in their life that related to the process of displacement: when they left home, when they became pregnant, when they were forced to leave their towns, and when they arrived in Bogotá.

**Results:**

Participants’ life stories showed how they struggled with agency during their lives. They were victims of abuse and violence during childhood and finally decided to leave their homes. They decided to have their babies despite the fact that they were abandoned by their partners and families, and after doubts about and attempts to have an abortion. Throughout the process of displacement the participants had been engaged in ambiguous relationships with armed groups. Finally they arrived in Bogotá and faced adverse circumstances but were looking for better opportunities for them and their children.

**Conclusion:**

The analysis of how internally displaced women narrated their life stories showed us that the concepts that dominate scholarly debates about agency, victimization and survivorship do not do justice to the life stories of the participants in our study. These stories show that changes with a major impact were loaded with ambiguity and were characterized by helplessness, lack of control and agency simultaneously. The reconstruction of these life stories goes beyond the stereotype of displaced women as only ‘victims’, but points also to their agency and courageous decisions they made in contexts that were not controlled by them and where support was often lacking. Instead of label them, it is important to understand the complexity of the life experiences of IDW, in order to build policies that offer them aids as victims, but also build policies and intervention programs that empower them as agents in order to support them during resettlement.

## Background

### Armed conflict and internal displacement in Colombia

Armed conflict in Colombia has a history of more than fifty years that still continues, despite the peace treaty signed by government and the biggest guerrilla group FARC (Fuerzas armadas revolucionarias de Colombia, in English: Revolutionary armed forces of Colombia). According to the Victims Record of Colombia, from 1985 to 2013 there are 2.683.335 women that are victims of the armed conflict. From those, 2.420.887 women have been forcibly displaced, 1.431 have suffered sexual violence, 2.601 have been forced to disappear, 12.624 have been killed, 592 have been hurt by an antipersonnel mine, 1.697 have been illegally recruited and 5.873 have been kidnapped [1].

Women have been described as the main victims of the armed conflict, especially in the Colombian cultural context that in some regions is still considered to be ‘machista’ and patriarchal one [2]. Cadavid shows how women in Colombia are vulnerable targets of the armed conflict, a condition that requires specific policies that aim to protect them [3]. Alzate points out how displaced women in Colombia are victims of all kinds of violations of their sexual and reproductive rights, such as the right to health, right to decide on the number of children, right to physical integrity and to live free from violence, and the right to privacy [4]. In addition, according to the Consejo Nacional de Política Económica y Social (CONPES, in English: Politic, Economic and Social National Council), there is visible recognition of the power relationships among men and women, whereby masculinity is considered as privileged, unequal and unjust [5].

On the other hand, some authors in Colombia have also explicitly stressed Colombian women’s agency instead of being victims only. Osorio described them as ‘survivors’ of the war but even more, he showed their impressive resistance to these extremely painful and precarious situations, positioning them as ‘agents’ who can restart life and overcome their displacement [6].

In this study we were interested in how Internally Displaced Women (IDW) living in Bogotá themselves articulate their experiences of victimization and agency. To that purpose, we studied how they reconstructed their life stories and reflected on their own experiences.

### Debates about victimization, survivorship and agency

Violence against women during and after armed conflict is globally recognized. United Nations reports explain how armed conflict exacerbates patterns of violence against women in different ways: increasing the incidences of everyday violence, particularly domestic violence, and increasing as communities break down during and after conflicts [7]. Additionally, among different kinds of violence, sexual violence in particular is used against women during and after armed conflict. As Haeri explained, sexual violence is used to punish, shame, intimidate, or simply to destroy the fabric of a community. Jack et al., explain that ‘By raping women, who represent the purity and culture of the nation, invading armies are also symbolically raping the nation itself” [8].

Violence against displaced women has been documented worldwide [9]. In Europe, for example, Freedman [10] showed how women refugees from Syria who arrived recently in Europe to find protection, are vulnerable to multiple forms of insecurity and violence. They have been forced to engage in sex to pay for their passage, among other violent acts [10]. In Asia, experiences of armed conflict and displacement in Syria, Sri Lanka and Nepal, clearly show how women experience violence [11–15]. Alsaba pointed out how even new forms of violence emerged and existing patterns of violence are often amplified and intensified as sexual violence. The author said that violence against women is understood as a tactic of war that includes military sexual slavery and forced prostitution in Syria [11]. Africa is not an exception, with examples in different countries such as Uganda and the Republic of Congo [16, 17]. Jacobsen and Dryden pointed out how refugees in these countries have been found to be at risk of sexual violence, human trafficking or labor exploitation [16, 17]. In addition, Knap pointed out how migration represents a drastic life change and gender roles and relations often shift in this process; this has been even more difficult for women [18].

Historically, violence is considered to be committed by men [19]. For example, Goldstein’s historical review pointed out how it is implied in history that men fight and women do not [20]. Women are portrayed as supportive to men when they go to war, in example providing them with entertainment and relief after battles while the ones who fight are the men. Furthermore, the author explained how the roles of women remained consistent after war, especially the responsibility for home life [20]. As Jack argued, the construction of the identities of women in their gendered roles as mothers and guardians of the culture implies that they are vulnerable and need protection [9]. This identity is embedded in culture as described by Elshtain in 1989. She suggested the features of women’s role in war as two opposites: the ‘beautiful soul’ or the ‘Spartan mother’. The first depicts women as better human beings, distanced from the dirt and brutality of the world, with the mission of being mothers and wives. On the other hand, the Spartan mother encourages men to fight, or wishes to fight herself, and actively supports war [21].

Many authors over time have agreed that in war zones, being a woman is a synonym of being a victim, just based on their gender. Some authors define ‘victim’ as any form of violence of abuse committed against women [19, 22]. This concept even initiated a specific research field in criminology, namely victimology [19, 22]. The use of the concept ‘victim’ has been controversial especially related to sexual violence and rape. Discussions about this notion reflect the tensions between the fields of feminist studies and victimology. Victimologists, working in a specific research field in criminology, prefer the use of ‘victim’ for persons who suffer a sexual assault, and feminist researchers prefer the term ‘survivor’ [19]. As Walklate explained, the use of the term ‘victim’ has been associated with blaming and re-victimization of the person who was sexually assaulted. The author also argued that the term ‘victim’ has implicit the assumption of a specific stereotype of ‘normal’ victim. The stereotype of a victim allows men to judge if the behavior of the victim was or was not appropriate, for example, dressing in a particular way that may incite male to rape [19]. According to Seririer, the concept of ‘survivor’ was introduced in the eighties in order to empower women to ‘speak out’. When women share their experiences, their speech permit them to recover their autonomy. They are not blamed or made to feel guilty. They become heroic activists and speakers, rather than silenced victims. It also is an inspiration for others who suffered assault to change their thinking around self-blame or isolation. They could understand that there are more survivors of this kind of assaults. It contributes to changing the social myths and victim-blaming attitudes about rape [23].

The use of the term victim or survivor is fundamental, because can be internalized and made part of one’s identity [24]. For example, the label ‘victim’ could imply several things. First, it could mean that the individual was passive or accepting of their assault and may not currently be actively working to overcome the situation. Second, it can theoretically lead to self-conscious emotions such as shame, guilt, and a lack of self-compassion: feelings which impede recovery from traumatic events. [24]. On the contrary, the term ‘survivor’ implies strength of will, resistance to the assault or self-shaming after-effects, and an active role in facing one’s traumatic experience and recovery. Survivor labels present the individual as an agent who does not experience abuse passively. In that sense, self-labelling as a survivor would result in coping better with traumatic events and experiencing positive mental health outcomes [24].

In addition, it might be a better strategy to frame oneself as a victim to stress innocence and vulnerability, for example, in the context of a court trial [25–27]. However, to recover from the experience and create a positive self-identity, adopting the term survivor might be the better strategy.

Although we found the use of the term ‘survivor’ to be very important, in this study, we focus more in the term agency, because we think that our participants expressed in their narratives not only experiences of sexual assaults (survivors), but they expressed how they passed though many other different traumatic experiences including armed conflict and internally displacement.

However, some authors have a different perspective regarding men and women roles in conflict and post conflict settings. For example, according to the Human Security Report, 2005, ‘With the critically important exception of sexual violence, there is considerable evidence to suggest that men, not women, are more vulnerable to the major impacts of armed conflict’ [28]. In this report, it is pointed out how males are more likely to die on the battlefield, but also more likely to be victims of collateral damage [28]. Moreover, Haeri explained how, far from being passive victims, ‘women from all walks of life including peasants and members of the educated class such as teachers, nurses, journalists and even nuns played a key role in sustaining the conflict and demonstrated their potential for inflicting extraordinary cruelty’ [8]. Examples include the atrocities committed in the genocide of Rwanda, where women participated actively as killers from the Hutu group and other women fought in the Tutsi resistance [29].

Cohen points out that men and women are likely to succumb to and participate in violent behavior under certain conditions [30]. She explains how combatants of both sexes may face enormous social pressure to commit violence and that both sexes are likely to respond to such pressures in similar ways [30]. For example, gang rapists where pressures from a group can cause individuals to behave in ways that they would never do on their own. Women and men are subjected to similar pressures from within armed groups and, facing similar circumstances, can be expected to commit similar atrocities. Moreover, she explained that, ‘women perpetrate wartime atrocities is surprising only because of the gendered assumptions that scholars and policymakers often make about women’s capacity to commit violence’. [30] Inspired by these studies, some scholars discuss the conceptualization of agency and victimization in zones of war, violent conflict and displacement. Hutchinson, in her literature overview reframes women from passive victims to active agents, able to draw upon personal strengths and resilience to develop strategies, which maximize survival chances. She also points to the paradoxical character of ‘passivity’ as a potential strategy of women to survive [31]. She observed this for instance in the case of North Uganda, where young women seek to marry or to become pregnant by high commanders due to the associated privileges such as exceptions from hard labor [16]. Some authors described how girls can use sex and marriage consciously and voluntarily to bargain themselves into units and to gain access to food, water and other material goods [8, 31, 32]. According to Heari, women show remarkable strength in coping with the challenges of living in war and often adopt new roles and responsibilities to care of their families and take part in community life [8]. In Sri Lanka for example, Rajasingham suggested that despite the psychosocial traumas that displacement entails, women found an opportunity for greater personal and group autonomy finding new spaces as heads of households or income generators in a post-conflict setting [13].

The term ‘agency’ is linked as well with resilience. Some authors define resilience as: ‘psychological attributes of an individual which may protect against negative consequences’ [33]. It is also conceived as the maintenance of healthy/successful functioning or adaptation within the context of a significant adversity or threat and is better characterized as a dynamic process, since individuals can be resilient to specific environmental hazards or resilient at one time but not another [34]. Nevertheless, subsequently the focus shifted towards exploring how broader systems facilitate processes of resilience. This led to an understanding that resilience processes are facilitated by culture and context-specific transactions between individuals and their social ecology [35].

Regarding the term ‘agency’, the debate goes further. As Hitlin notes, ‘Agency remains a slippery concept because of inconsistent definitions across theoretical projects’ [36]. For example, Campbell [32] defined this debate as the ‘black box’ of ‘personal agency’. In his essay, he identified two main conceptions regarding agency. The first one, agency is defended for some authors as ‘the power that individuals possess that enables them to realize their chosen goals’. Against this position, some other authors, such as Weber [32] defined agency as ‘the probability that one actor within a social relationship will be in a position to carry out his own will despite resistance’. Salzman argued that the concept of agency includes the intention, options, purposes and how people could achieve their goals, but in a context of social norms that they are not following passively, but modifying their social worlds [37]. Hitlin also explained, that agentic behavior is influenced by the requirements of the interactions; as actors become more or less concerned with the immediate moment versus long-term life goals, they employ different social psychological processes and exhibit different forms of agency according to the self and the time [36].

In their book: “Beyond Mothers, Monsters, Whores: Thinking about women’s violence in Global Politics”, Laura Sjoberg and Caron Gentry, pointed out regarding agency, that people do not make choices independent of either other people or the social structures around them. They argued that people do make choices, but that those choices are both heavily and differentially constrained: “By heavily constrained, we mean that a wide variety of social structures, expectations and significations play a role in constituting conditions of possibility for choices and the choices themselves. By differentially constrained, we mean that both the level and type of constraints differ across people’s positions in social and political life – based on gender, race, class, nationality and other features of position in global politics’ [38].

Inspired by these scholarly discussions, this paper will explore the different tussles between agency and helplessness that internally displaced young mothers in Bogotá lived, according to their life stories and their own perspective.

## Methods

### Study design

This research was qualitative in nature and took an ethnographic approach, seeking to learn from the life stories of young mothers how they experienced agency and helplessness before, during and after forced displacement. Alongside that, we organized workshops with IDW in the community, which enabled a process of sharing and exchanging experiences in a safe atmosphere and to collectively reflect on the issue of victimization and agency.

### Study setting

This research was conducted in Ciudad Bolivar, a locality^1^ in Bogotá, Colombia. This locality is the one that receives the majority of Internally Displaced Persons in the capital [39]. The 94% of people who live in this locality are in the lowest socioeconomic status. This locality is one of the most violent of Bogotá, with a high incidence of murders and violent acts [40]. In this area, there are some members of the illegal armed groups, even guerrilla or paramilitary [41–43].

### Data collection

The first author, YC is a female physician-anthropologist who worked there since 2006 as part of the social extension program of the Pontificia Universidad Javeriana, known as the Vidas Móviles Project. That project wanted to help internally displaced families in different areas such as health, housing, and life-conditions. The fieldwork for this research was developed between June 2015 and May 2016, a period when the first author visited the locality two or three times per week, and irregularly (twice per month) in the following two years. During the first year of fieldwork, YC who was familiar with the research context and had experience in the field, collected the life story narratives through in-depth interviews from twenty internally displaced women who were pregnant during adolescence. In addition, YC organized eight workshops with the same twenty women who were interviewed, but also with another fifteen women from the neighborhood. All of them were invited to these workshops as taking part of interesting activities provided by the project for the neighborhood. It allowed participants to elaborate freely on their experiences and their ideas about topics that were discussed in the interviews. These workshops developed these topics through creative processes such as painting, writing, writing on the wall, singing, and role-playing. The last two workshops provided were developed in addition to provide feedback of the results of this research. Six of the workshops took place in the project building, and the last two in two houses of participants.

Participant observation was made by the first author during the visits conducted to the neighborhood, taking notes about informal conversations in the daily life activities with people form the community including visits to some of the houses of participants.

### Sampling

Participants were twenty IDW, all living in the locality, who were mothers during adolescence, and Their age at the time of this study varied from 18 to 35 years. We choose the definition of adolescence according to The World Health Organization, that is ‘young people between the ages of 10 and 19 years’ [44]. Most of these mothers had their first child at the age of 14. They had one to six children.

As the first author has contact with people from the neighborhood, we chose key informants to invite different participants via a snowball sampling strategy until reaching saturation point [45]. The inclusion criteria for this research were: women who have been forced displaced and have been mothers during adolescence. The majority of participants were part of the program, but it was not an inclusion criterion. For example, to be part of the project, participants need to have had the status of displacement provided by the government, that was not the case for participants of this research. The project had different family members including fathers and boys, that was not the case of this research.

All participants were interviewed by the first researcher individually in Spanish once, twice or three times, and all the interviews were recorded. All interviews were open, giving participants freedom to talk about their life stories.

### Data analysis

To analyse the data, all the life stories, interviews, and workshops were transcribed in Spanish and translated in to English.

We analyzed the narratives of their life-stories from the moment of their childhood in the countryside to their situation at the moment of the interview in Bogotá. For this study, life story is defined in accordance with Pujadas: ‘It is a story about the self-life, which is obtained by the researcher through different interviews. The objective is to show the subjective testimony of a person. It includes the experiences but also the perceptions that that person made of his/her own existence’ [46]. The objective is to understand all the aspects of that life, and it is incentivized by the presence of a researcher. It means that the story is a result of all those interactions [46].

We use the comprehensive analysis defined by Bichi, 2002, that takes in to account the life cycle, the experience and the interview itself [47]. We started with familiarizing ourselves with all narratives and subsequently we analyzed the narratives on specific themes meaning the moments of their lives when they showed agency, or were survivors or victims. As Charries explains, the biographies try to cover time and place form childhood until the moment the life-story is narrated, including different aspects of the self. They allow as identifying the ambiguities and changes, as a vital process, and the critical moments in the individual. They permit as well to identify the subjective perspective of the individual, as well as the interpretation of their own acts. Biographies permit to understand social phenomena that only could be understood though the personal experiences of the concrete individuals. [48]

In this article, we present an analysis of four life-stories that, besides the similarities that those cases represent regarding rural origin, armed conflict context, and teenage pregnancy, show as well the diversity of experiences that could be followed during a lifetime. We selected those particular life-stories primarily because of the way each one deals comprehensively with the maneuvers made by victims-survivors over a lifetime. We will follow their narratives from their childhood in hometown to their settlement in Bogotáin the four moments as turning points or transitions in their lives. It provides a better understanding of how they deal with agency and helplessness during the same life. Those were the moment they left home, when they became pregnant, when they were forced to leave their towns, and when they came to Bogotá as IDW.

The analysis was regularly discussed with the other authors. In order to receive feedback regarding the adequacy of the interpretations, the analysis was presented to and discussed with the participants in two workshops.

### Limitations

As a qualitative approach, the empirical findings cannot be generalized to other populations in other contexts. The focus on life-stories limits an in-depth focus on a specific theme. Nevertheless, the conceptual analysis can provide important insights that may be helpful to understand difficulties of displacement in other contexts.

People from the neighborhood known the first author as a physician and as part of the project Vidas Móviles. Participants in the study expressed that condition as a plus, because they trusted the researcher and felt that they could share their experiences, including medical issues.

Eventual bias is also reduced because all data and interpretations were discussed with the other members of the research team, who were not part of Vidas Moviles.

### Ethical framing

Research and ethical approval was granted by the Ethic Committee of the School of Medicine of the Pontificia Universidad Javeriana, Bogotá (Act 21/2015.FM-CIE-8744-15). In the same way, research protocol and methods were consistent with the Colombian Law. All participants had their first baby during adolescence, but at the moment of the interview their age was between 18 and 35 years old. Participants were asked to provide and to sign informed consent. We took care that the participants were fully informed about all aspects of the project and were aware that they could withdraw at any moment without providing reasons, and that eventual withdrawal would not affect their relationship with the community project Vidas Moviles in any way. Recordings were destroyed, and transcripts will be stored in accordance with Dutch Law in a protected location at Maastricht University for 10 years.

This research followed the ethical recommendations from the American Anthropology Association and continuously reflected on possible ethical concerns beyond informed consent during the study. We protected the identity of the participants by changing their names in the text. In addition, we did not identify the violent groups that are mentioned as causing displacement. We took care of the participants by following up during monthly workshops and helping them with some difficulties they were facing (e.g., helping them to access the aid offered by the government, finding schools for their children, or attending to certain questions related to health).

## Results

In this section, we will present fragments of four life stories, which show how they reconstruct four important moments in their lives in terms of agency and helplessness. These four moments were when they left home, when they became pregnant and had the baby, when they left their home towns, and when they came to Bogotá as IDW.

## Leaving home while still young

For all of our participants, childhood was shaped by violence and mistreatments at home. The diversity of the stories explain difficult childhoods, some of them without fathers, because they were in jail or killed because of the armed conflict. Other participants lived with stepfathers, or other family members, but all of them expressed living with violence and having sad memories from their childhood. Their narratives described the struggle to stay at home, until they decided to leave. One example is Vicky who was thirty-five years old at the moment of the interviews. She was born in a town from the south of the country and had one sister and two brothers. She had five children with three partners. She told us about the difficulties in her childhood and her decision to leave home.

‘When my father passed away, my mother left me with my grandma. Grandma was nice to me. She hit me, but because she was teaching me to do things … you know. She was old. I was with her until I was nine. And she passed away, so I had to live with my aunt, Ursula. She was very bad with me. She did not like me. She hit me a lot. She left me without food, locked in the house. And the neighbor prepared big pans of food… I started to steal the food from them. I was thinking about how to leave that house. My older cousin tried to rape me, and I was too tired to take care of my little cousins, so one day, I decide to break the hand of the little one. I knew that if my aunt arrived, she would kill me. I decided to escape. I was used to doing it through the back window, to go to my neighbors, so I did it. I spent the night hidden there, and the next day, after eating something, I escaped from my neighbor’s house. So I started to walk, and a police officer asked me about my mother. I explained everything to him… and he decide to take me to the Welfare Institute. I lived there a couple of years’ (Vicky).

The Welfare Family Institute is a government organization that take cares of children who do not have families or have families that cannot support them properly, in terms of food, care, or health. In the next phase, she again made some bold choices. Vicky, as with all our participants, suffered from different kinds of violence during childhood, by parents and caregivers. She expressed how she ‘was thinking of leaving that house’ and finally she ‘decided to escape’. She explained that when she was at the Welfare Family Institute, one couple wanted to adopt her, but she refused, because she did not want to be ‘given’ to others. So, in a very violent context, she made difficult choices to take care of herself, in accordance with her own ideas, and she resisted that violence at home until she left it.

Other stories also showed how women made choices to escape sexual violence. Esther, in her life-story, is not an exception. She was a woman of twenty-six years old, the second of seven siblings. She left her home when she was ten. At the moment of the interviews she had three children. This is what she explained us about her childhood:

‘I was born in a small town in a central department of Colombia. I lived with my six brothers and sisters… but we were not so close because four of them were from another father. I was from the first husband of my mother. I used to live eight days with my mother and eight days with my grandmother. The husband of my mother gave us a terrible life. He hit my mom all the time, until he killed her some years ago, when I was pregnant. I was suffering a lot with them, so when I was ten. I started to work for a person, doing the cleaning, cooking and helping her. It was also difficult with my grandmother, because there my uncle raped me. That was also a reason I left them. I told my mom about the rape, but she never did anything. So it was like that’ (Esther).

In both narratives, we see how the women presented their experiences as victims of mistreatment at home, but also how they resisted this mistreatment and at one point decided to leave home.

1. Becoming pregnant and having a baby

To have a baby and become a mother is a life changing experience. For all participants, the first pregnancy was at an early age, unexpected and not a deliberate choice. In the case of Vicky’s narrative, the first pregnancy was at thirteen. She explained that she was living with a friend and she fell in love with the father of her two first children:

‘I was thirteen, when I got pregnant. I was living with my friend and I met the father of my oldest two. At the beginning, everything was nice, the men took you to the park, and picnics to the river, and dancing. But later, they change. When he realized that I was pregnant, he changed completely. He was twenty-eight and I was thirteen. He locked me in the room, very possessive, jealous…’

I: ‘And you knew about family planning?’

V: ‘Yes. I had gone to the hospital, but they said that I was very young to do it… and after the first baby, when she was one year old, I got pregnant again.’ (Vicky).

It should be noted that Vicky knew about family planning methods, but according to her interview, health professionals at the hospital refused to give her any method, because of ‘her age’. She explained and believed that her pregnancies were also result of the lack of support of the health professionals. She tried, but the guidance regarding family planning was denied. Referring to this she considered herself a victim of the health care system. The two pregnancies were unexpected, but she explained that she was in love with the father and continued living with him, even though he mistreated her. She clearly identified herself as victim of inter-partner violence. For several participants, the romantic trajectories were spoiled by the mistreatment of their partners after some time. This common experience usually ended because something happened with their partners, they were killed, or went to jail. Vicky related that she continued living with her partner and their two children until her husband went to jail for two years because of theft. Vicky lost the third pregnancy because of a big fight with her husband. Some months after, her husband was killed. She pointed out that it was a very difficult moment in her life.

Similarly, Esther evoked her difficult pregnancies. In her narrative, she expressed how all the pregnancies were unexpected and hard:

‘… I started to hang with friends, and I met the father of my children. I met him when I was sixteen and I got pregnant at eighteen. It was an unwanted pregnancy. I was alone during the pregnancy, because the father of the baby went to the army. He decided to leave me. But after the delivery he came to see us… My daughter was born sick… I think it happened because of the death of my mom during that pregnancy. Her husband killed her. I was shocked… I found her killed, in the living room, smelling very bad (crying). Later, I got pregnant again. It was also a surprise, because I did not want it. But my husband was happy, because it was a boy. And then, when the baby was three months old, I got pregnant again. I was not using any method…. And then I noticed that my husband had another woman. But then, he asked me forgiveness… and what can you do with three children? So I accepted him. And then after two years, he went to jail. And he left me alone with the children’. (Esther)

Here Esther’s narrative reflects how she openly chose her partner, and how she did not use any method of contraception even after her first pregnancy. However, she explained how she was abandoned by her partner during the pregnancy, and later she struggled with the relationship with her partner because of his infidelity. Additionally, she referred to herself as a victim of the violence of the stepfather, who killed her mother. She also presented herself as a victim of the circumstances that did not permit her to take care of her siblings.

Mary’s pregnancy was unexpected too. She told that she became pregnant when she was fourteen. She shared how she was struggling with the idea of an abortion, but that she ‘decided’ to keep the pregnancy and become a single mother.

‘When I started to have sexual relationships with Mark, I was fourteen years old. He is pretty much older than I am and when we had sexual relationships, he asked me if I took the pill. I answered him, “No, because I don’t have headache”’ (smiling).

I: ‘That means that you did not know…’

M. ‘No!. And when I knew about the pregnancy… Oh My God!… I was the one who walked away from him. I did not want him. My brother looked for him, but I took the decision. My pregnancy was very difficult, because I had preeclampsia. I was afraid, so I told my mom that I was pregnant, but she reacted very badly and started to ask me why… “You are very young! And how many months of pregnancy do you have?”… and I had already four months… I tried to abort… that is something that hurts so much (crying)… I decided to have him alone. My mom later wanted to take my son, but I said not to, because it was my responsibility’ (Mary).

In the story, becoming pregnant unexpectedly on a very early age is related to feelings of lack of agency and control. Nevertheless, phrases such as ‘I decided to have him alone’ indicate how she determined to make her own choice with respect to continuation of the pregnancy and to resist other opinions as her mom’s. She also ‘took the decisions’ to not tell her partner about the pregnancy and to leave him.

Carla explained how she got pregnant unexpectedly, and she did not know of contraception. She also explained how she was struggling with the idea of abortion as well and actually tried, but following her mom’s advice, she changed her mind.

‘I did not know anything about family planning… My mom did not explain me anything about that… At the age of fifteen, I had already three months of pregnancy. I just stayed with a boy!… I thought that if I said anything my mom would kill me… but that month I didn’t asked my mom for sanitary towels. So, she slapped me, because I was taking something to abort (crying). So, that was hard, but I think I could understand many things… So because of the pregnancy, my mom took me to the farm with my grandpa and my uncles. They were not nice at all. I worked in the coca crop, but I was bad at that, so they moved me to the chemicals, but the gasoline made me dizzy… so I was moved to the kitchen, and my uncle made fun of me “That is the thing you wanted! Why you are pregnant!” Once I was milking the cow, and I received a kick… I thought I would lose the baby.’ (Carla)

The fact that Carla decided to keep the pregnancy following her mom’s reaction might be related to the conservative Colombian moral culture in some regions that considers abortion as illegal and as a sin. However, she described how she was verbally mistreated by her family and had to work during pregnancy in bad conditions. Regarding the partner, there were many expressions in the interviews whereby women took for granted the way they felt they were in love and became pregnant. In one example, Carla also mentioned that ‘the flesh is weak’ when she was sharing with us about the relationship with the father of her second child:

‘When my first baby was six months old I came back to the village. I helped my mom to prepare food to sell. And you know, the flesh is weak, so I met another boy and just happened. I had two months on pregnancy, when I started to feel bad, so my mother took me to the nurse. I told her with a very low voice “I am pregnant”. And suddenly she said it to my mom! My mom started to ask me, “Who is the father?” and I said, “Who cares! It is only mine!. (Carla)

The life story of Carla reflected pride about her pregnancies and about having a baby for herself, on her own. She pointed out how she resisted family or society’s opinions regarding her pregnancies. Even more, in relation to the abortion, Carla’s narrative pointed out how in her third pregnancy she struggled to keep the pregnancy, despite diverse opinions of family members, society, her economic situation, or the bad relationship with her partner. She said that she was working in a house for a lady, doing the cleaning. She was twenty-four.

‘…The lady said to me that I cannot have more babies and that if I was pregnant I could not work anymore… But I worked harder. My brother also told me, “you are pregnant again! What are you going to do? It is not possible…” He was suggesting for me to abort… but I told him, “don’t be silly, I didn’t abort the others, I will not abort this one!” (Carla)

Several participants had an ideal of a harmonious and happy family. Their narratives showed the desire to be married to a good partner and in the context of this ideal they sometimes agreed to have another baby to please their partners. Carla had four or five children from different partners. Her story reveals the struggle to take the decision to have another baby in order to build a family with her new partner.

‘My fourth child was a miracle. I was starting my relationship with my current husband. But because I already had other three children from different fathers, I had asked for the surgery to not have more babies. After some time, I started to be dizzy, and I thought that is not possible… But at that time, I wanted to have another baby… So I went to the doctor, and he said “you want another baby, but it is impossible. Anyway, I will ask you to take a pregnancy test, and don’t bother me anymore”. My husband wrote me a letter as a gift, you know, that he wanted a baby, so that day I prayed to God, because you know, you like to have a family, a beautiful family… I am not a bad woman, and I really hoped to have a home, with a husband and children! So I asked God, but for a male, not a female, because females… we suffer a lot… and then, God gave me a girl!’ (Carla)

Carla’s story conveyed the desire to have another baby. She prayed, she went to the doctor, and she asked for a miracle. She resisted advice against this wish, including the doctor’s.

## Engaging with armed groups and escaping

In general, participants described their rural environment as aggressive and violent due to the armed conflict. Some areas in Colombia were suffering due to the guerrillas, the paramilitary groups and sometimes the army. Participants described that this environment forced people to choose and to be part of one or the other group as a way to feel safe. The stories show how the women became involved in the war, as they had family members or partners in these armed groups, and they even became part of an armed group themselves. Continuing with the narrative of Vicky, she explained that during this specific crisis, she received an offer to work as a cook, on a farm. However, that appeared to be a false setup. As some authors have described, the guerrilla or paramilitary groups took girls from the countryside, cheated to be part of those illegal armed groups, and asked them for sexual relationships or to be victims of human trafficking [1, 3]. This was the case with Vicky and her cousin.

‘I really need the money because my children were in the Welfare Family Institute because I didn’t have the money to take care of them, and I wanted to be with them… I was twenty… so my cousin and I met a woman. She told us that in Guaviare her husband needed women for cooking and that it was a very good paying job. So we wanted the jobs… I was thinking six hundred thousand pesos is a lot! So I wanted to do my best, so I prepared myself, I did my hair and nails, in order to be accepted in the job. She paid our tickets and we took a flight to Guaviare, spending one night in Bogotá. She paid everything and said to us that we can reimburse her later. When we arrived, there was a tall man who said, “Hi, I am the Commandant Lucas, these are fresh flesh…” And he took our bags and we went in to a house with girls without clothes… a prostitution house. I was shocked, very afraid and I said, no! We came here to work as cooks. He started to laugh and he said that we cannot go until we paid the flight tickets and the hotel… I was very beautiful, because I had prepared myself for the job, even though I was poor; I wanted to be prepared, with my hair and nails… My cousin was fourteen, and she had big breasts so many men wanted to be with her… then the commandant said to me, “I will be with you, black girl, tonight, the whole night…”’ (Vicky)

This narrative shows how the women become victims of sexual violence from the armed groups. Vicky wanted to resist entering the prostitution house, but she was unable to. After being cheated into the guerrilla and having been victim of human trafficking, she had a partner who was from the guerrillas, and he was the father of two more children. This way she became part of the armed group. She explained that after some time, when she decided to leave, she literally escaped from the two-armed groups. Here Vicky reflected that experience:

‘I was in the guerrillas, but I got sick… so when someone is sick they don’t like that person to die in the camp, so I left the guerrillas, and the father of my children, who was an *armed group 1* man. I found a job on a farm, with an old man, but then he wanted to have sex with me, but I didn’t like him… so I refused… And that stupid old man went to the *armed group 1* to tell them that I was now from the *armed group 2*. So one day, some of the *armed group 1* arrive in to the farm and told me: “Come here, *armed group 2* bitch!” and they hit me, and I started to cry, and shout, “I am not *armed group 2*! How many times did you have lunch in my place, and I gave you my hens and my food! I am not an *armed group 2*!” And they continued hitting me… some of them were digging my grave… I thought only of my children… I begged them: “I am only a cook, please, don’t kill me”. So, one of them went to find the father of my children… so they decided to leave me there… At midnight one boy from the *armed group 1* arrived at my home, and he told me: “They will come to kill you at 6 a.m. You have to go, now”. So I packed some clothes, and took my two girls and my baby, and I started to walk, I heard any noise and immediately I was trying to hide… and it is difficult because you need to be aware of snakes, and the mud… When a group arrived, I thought, “We are dead… is it the *armed group 1”*, so they asked me: “Lady, where are you going?” And I understood that was the army: “Are you slipping away?” And I answered: “Yes”. So they told me, don’t worry, we can help you and lead you to a safe place…’ (Vicky)

Vicky explained how she and her children were escaping from one armed group, she received some help from the army, and she continued explaining how they met the other armed group. She shared how they were in the middle of the different armed actors in the village.

‘So we continued walking for three more hours… but then one fight started, and they shouted: “Lay down! Lay down!” And there were shouts everywhere… My children and I were on the ground, with mud and cow shit, terrified… because they also started the bombs from the helicopters… So we continued until the river… The army asked a person with a small boat to lead me to the next village, but we need to be hidden. So they put us under rice sacks and we started to move… It was very hot, and without air… My poor children… It was very difficult… then, another fight started… it was the *armed group 1*, so they stopped our boat, and they asked to the driver: “Do you have the *armed group 2* bitch? We are looking for her to kill…” but the driver said, “Comrade, I only have this rice to sell in the village.” So they allowed us to continue. After some kilometers, the driver let us put out our heads out, and I saw the border of the river! It was a relief… but then I saw one of the heads of the *armed group 2* there… He stopped two boats, and he said: Finally these son of a bitch *armed group 1* people… And he asked us to leave the boat. So I was so nervous and I started to shout: “I am not a *armed group 1*, I am escaping one day with my children from them! I am not!!! Kill us now! I am upset with this shit!” So, the man told me, “don’t shout, because I don’t like that”. So he put me and my children in the right side, and all the other people in the left. And he killed all these people in front of us… And then he let us go….’ (Vicky)

The story of Vicky counters the image of weakness and emphasizes the courage of displaced women. As Vicky recollected her experiences of displacement, she emphasized her desperation to save her children and herself from the war. This intense wish for safety drove her to face the power of the heads of the armed groups. Finally, Vicky expressed that she decided to come to Bogotá, looking for better economic opportunities and better health. In her story, she presented herself as a victim, but she also resisted all the organizations of armed groups in order to recover her freedom, start a new life and provide better opportunities for her children.

The relationship with the armed groups, in the story of Esther is explained when she was an active member of one armed group. Her husband and her were in charge of taking care of kidnapped persons. For this reason, his husband went to jail.

‘We worked on a farm but my husband helped the *armed group* keeping kidnapped people… and once, one of the kidnapped was killed. When I was pregnant with the third, people from the prosecution office took my husband and put him in jail for 25 years. After my last child was born, people from the prosecution office took me as well… but I did not do anything…but they accused me of being an accomplice, that I knew everything… and it is true, but I could not say anything to the police or others… you know, *they* (armed group) would kill me… so I told that to the Prosecution, and then I was free again. So I came back to the farm, but two days later, the *armed group* arrived, they wanted to kill me because they thought that I had gone to the Prosecution to tell stories about them and about the murders and kidnappings… and that I was responsible for the prosecution findings… And I told them: “That is not possible, because I need my husband, and I never would go to accuse him or you in the Prosecution” but they did not believe me… So, they told me that they would kill me if I stayed on the farm… They gave me two hours, so I packed some clothes and asked my sister for money for me and my children for the bus…’ (Esther)

These examples show how women were victims of armed groups, but in this role also took part in the armed conflict. Some of the participants explained that they knew about kidnappings, murders, massacres, or coca crops, but that they were involved because of their circumstances. Several studies have shown that due to the armed conflict context, some families are forced to take the side of one group or the other [1]. However, on many occasions, our participants mentioned that they chose one group to be part of as this group offered relevant help in that context. Sometimes, they made agreements with armed actors and sometimes they married armed group members.

The relationships of participants with armed groups could be very close, because some family members or partners could be part of these armed groups. Sometimes, women agreed with the ideology of armed groups and were proud of their partners or family members who were part of those armed groups. Mary, for example, conveyed her admiration for her husband, one of the heads of one armed group:

‘My son was eleven when I met Joseph. I met his family, so we began a good friendship, and we liked each other… so we started to live together. After one year, I got pregnant with my second child. We lived there for thirteen years… there I understood that Joseph (husband) was part of them (armed group). He was a communist and he did not find a place. The armed group took him when he was twelve years old, and when he escaped he was 21… he is amazing… chavist, fidelist… (Smiling) he is awesome! very serious, he can assemble and disassemble a weapon with closed eyes!… then, people from the army arrived… I thought, and then I understood that was the *armed group* … Joseph is a complicated person…. He says that there are no trustworthy people. He does not believe in the government or the State. He doesn’t believe in anything… on the contrary, I like to help people, so he arrived and there were a lot of clashes with the two armed groups… paramilitary people, and the guerrillas, and one of them offered him work, but he was with the others… Many of them were radicals before, they fought following an ideal, but today it is mixed with drugs, narco-groups. I know that Joseph is not a bad person… he just passed through many things… we talk, we chat, and he knows all the things I do here in Bogotá.’ (Mary)

Mary expressed her intention to choose her husband and her decision to support one armed group. This choice however led to her being a victim of the violent context in the village. She shared that she lived in a village controlled by one armed group and how she lived through the massacre when her village was taken as the enemies of the other armed group.

‘We usually go to the market on Wednesday. But I remember very well that Wednesday… I will not forget that time, I was in that massacre, I was with my child and my husband on the boat, but the atmosphere in the village was different. Nobody said anything, all the people were looking at the floor. I understood that *they* (enemies-armed group) were in the village. I cannot forget that smell of blood and brains… My husband only said me “just walk… just walk…” but it was really hard. I started to see the corpses, the brains on the grass… I can’t not forget that smell… My child started to cry, but I couldn’t be there for him, because I was shocked… and I could not cry until we arrived at the house of my father in law…” (Mary)

Participants frequently became emotional during the interviews, demonstrating how much this civil war still affected them. Mary was still shocked in the moment of the interview and found it painful to remember that massacre. She said that she still had nightmares and depression. That was the reason to struggle with the decision of stay or leave. Staying meant being in danger because of the conflict, but leaving meant starting a new life, without anything, no money, no job, no family, and with children.

## Becoming displaced

Once the women arrived in the big cities after displacement, they faced many barriers in terms of access to housing, jobs, and health services, among others [4]. But at the same time, they had the responsibility of raising their children. Vicky explained how difficult it was to find a job and to take care of the children when she arrived in Bogotá. She foregrounded her feelings of sadness and depression.

‘Sometimes is difficult, because I need to eat and give some food to the children. Sometimes I wake up and I would like to be dead, because I feel very stressed… Sometimes I am angry with my children. I know they do not deserve it, but it is difficult. Sometimes I work in cleaning…’ (Vicky).

Esther expressed that it has been very difficult, but she was able to receive some aid provided by the government for displaced population. She also mentioned that her daughter with the disability is at the Welfare Institute. Esther presented herself as a victim of forced displacement, but actively searching for help and at some point she received aid from the government.

‘Here it has been very difficult. My husband has to spend twenty-five years in jail. Everything you do in your life, you must pay. But here in Bogotá, I have received some help. My children are at school and they receive food there. The oldest now is in the Welfare Institute. They took her away from me. I had to work, because of the food, so when I arrived home, she was dirty, and sick… So, they took her away from me. Now we live in a small room, and I work hard.’ (Esther)

The story of Mary reveals that she suffered three displacements in total. Nevertheless, at this moment in the fieldwork, Mary and another woman were recognized by the community as leaders and are considered examples of how to overcome violence, displacement and abuse. Mary was working in the community, helping other women who had newly arrived from the countryside and did not know how to proceed in the city or how to get access to government aid and health services. However, Mary shared her feelings of frustration and depression, which reflected how much violence and displacement affected her life, despite her leadership in the community.

‘I know how hard it is… I came from starvation, violence, and many memories that I would prefer not to have in my mind… I can understand them. I know what it means if you do not have any food to give to your children before going to bed… life is hard. I passed through many, many things, rapes, murders of family members, and my husband… I know how difficult it is to start your life again. Now, I feel so happy when I can do anything for others. Sometimes it is very difficult, because there are a lot of needs… Many things that you cannot solve… But I can confess to you than my heart is still in pain… sometimes it is like the clown… I need to smile and tell them that will be fine, but you still have your heart broken… I usually cry a lot.’ (Mary)

These feelings of depression and sad memories were present in all the interviews. In the last two workshops we organized a conversation about this study and asked for feedback on our interpretations. We told them that, based on the analysis of the stories, we considered them to be strong women, who had faced difficult situations but who took decisions and overcame those difficulties. Nevertheless, they explained that their decisions were forced by their circumstances. Moreover, they openly expressed that they described themselves as victims who needed to elaborate those experiences. They mentioned that they needed to put aside the mask of being strong women, and receive all kind of help. They showed appreciation for the workshops we conducted. They expressed that the workshops had helped them significantly. They expressed that workshops were an opportunity to share with others who passed through similar situations. They valued the workshops as opportunities to remember the experiences they have had and to elaborate on them in areas such as forgiveness, self-esteem, and to look to the future.

## Discussion

Against the background of scholarly debates about agency, victimhood and survivorship of internally displaced women, we analyzed life stories of internally displaced women with adolescent pregnancy in Colombia to understand how women themselves present their struggles with agency and helplessness. From the analysis it became clear how childhood was shaped by mistreatment and violence at home, leading them to escape the family home, to resist family violence and to take the decision to leave, despite all the challenges that this decision entailed. Secondly, the analysis of the stories shows that the pregnancies were unexpected, but that the women decided to keep the babies despite advices to terminate the pregnancy and despite a rather fragile and insecure relationship with their partners. Thirdly, the description of their relationships with armed groups were diverse and complex. The stories present the women as victims of armed groups who were stolen, cheated, or abused, but also as active members of those groups and sympathizers with their ideologies. Finally, the ways in which women narrated their experiences of displacement and resettlement in the big cities represented how they resisted the power of the armed groups and took decisions to leave the towns. They faced new challenges of displacement, and tried to adapt to the receptor community.

Many scholars have studied displaced women in terms of victims of the civil war. It is known worldwide how women have suffered the consequences of war, sexual violence and gender violence in those settings [10–19, 22]. In the specific context of Colombia, Meertens, for example, [49] argued that displaced women could be considered as victims three times: first through the trauma produced by the violent acts such as murders (partners of family), second, loss of their land (house, crops, animals), and third, the social and emotional uprooting from a rural area to a big city [49]. Cadavid showed how women are simultaneously intimidated within a machista and patriarchal society and main victims of war. [3]. Our analysis partly confirms these literatures. Internally displaced women do present themselves as victims of violence at home, of inter-partner violence, and of violence by the armed groups.

Other authors reframed women from passive victims to active agents [8, 16, 31, 32], developing resilience, resisting violence and taking decisions in order to maximize survival chances or even being active members of violent groups in war [29]. Meertens [50] illustrated how displaced women in Colombia were widows of rural violence, heads of household in the new cities, spouses, and leaders whose experiences of participation and organization help them to forge new life projects, individual and collective, in the city [50]. Following Campbell [32] in his essay about the ‘black box’ of ‘personal agency’, and the definition of Weber, ‘the probability that one actor within a social relationship will be in a position to carry out his own will despite resistance’ [32], the life stories can be seen as expressing agency.

Our analysis of the life stories shows how women escaped family violence at a very early age, which can be considered a brave act that expresses the wish to resist violence and to find a better place to live. They related to armed groups that they met while escaping home, but also decided to escape them again, as they did not offer the protection they needed. They decided to keep their pregnancies, not to choose abortion, although some family members or others advised them to do so. In many respects, the life stories testified to agency.

Our analysis suggests that the conceptual dichotomy of victims-survivors/agents is not a very fruitful way to grasp the life stories of displaced women, as it does no justice to the dual and ambiguous character of their experiences and the choices they made.

Laura Sjoberg and Caron Gentry explain, in their book about ‘Women’s Violence in Global Politics’, that categorizations of women that help researchers or policy makers, often do not attune very well to the everyday life worlds of women. It says, “In our enthusiasm to highlight what we saw as the most problematic element of the mother, monster and whore narratives, though, we discussed agency and lack thereof as a dichotomy, with a preference for the recognition of agency, even if that agency is a complicated, relational decision-making framework. In so doing, we related the recognition of women’s agency to the subversion of gender oppression, arguing that in order to ‘move towards a more gender-equal inter- national society, we, as scholars and political actors, must be willing to embrace and study the agency of not only the best of women but also the worst of women’. We argued that the path to this gender equality was through questioning scholars’ ‘own implicitly racialized and sexualized discourse in order to transform the (increasingly subtle) discursive’. [38] That the dichotomy victim/survivor/agent does capture the experiences of displaced women also became clear in our study. The life stories contain elements of both. Moreover, in reflecting their life stories participants explicitly underlined that whatever agency or resilience or courage they demonstrated, they felt that they were forced to take those decisions because they did not have other options. Moreover, survivorship also entailed that they still were struggling with depressive thoughts, memories of violence, lack of perspectives for the future, and with raising the children in a way they would have liked to [19, 23].

To conclude, while notions of victimization and survivorship can be helpful to guide the analyses of displaced women, our analysis of the life stories shows that the categorization of women as either victim or as survivor/agent does not grasp the ambiguous and dual nature of the experiences and reflections of displaced women.

## Conclusion

Our study shows that women themselves explain their life stories not solely in terms of victimization, nor solely in terms of agency. From the analysis, it is clear that the different experiences they passed through in their lives were not black and white: victims or agentsIt is possible to see how women in their everyday life were victims, but also survivors and agents.

The analysis of how internally displaced women narrated their life stories showed us that the concepts that dominate scholarly debates about agency, victimization and survivorship do not do justice to the life stories of the participants in our study.

We think that in the case of our participants in this study, the dichotomy of labeling as victims or agents/survivors remains incomplete, as the life-stories and the reflections of women show that they experience themselves as both. It may help to underline the resilience, the agency and the survivor skills of women as they display tremendous capabilities and sources of empowerment. On the other hand, women made clear that this may disregard the violent context and the fact that they felt they had no choice: ‘I was forced to do it’. Furthermore, women stressed that they were victims. Based on this, we belief it is important to develop policies that do not require overly simplistic indications for help and that do not force women to label themselves into one category. Policies should enable help and support for victims without denying their capabilities to re-build their lives during resettlement, and to make their own choices. In administrative policy practices, with a tendency to make clear categories and indication, this may be difficult, but this should be the point of departure. After having escaped violence, women should not be forced to present themselves as either victim or survivor. Moreover, policies should empower them as agents and brave women that they already are, in order to support them during resettlement.

## Abbreviations

CONPES: Consejo Nacional de Política Económica y Social (Politic, Economic and Social National Council)
ELN: Ejército de Liberación Nacional (National Liberation Army)
FARC: Fuerzas Armadas Revolucionarias de Colombia (Colombian Revolutionary Armed Forces)
IDPs: Internally Displaced Persons
IDW: Internally Displaced Women
PA: Pregnancy in Adolescence

## Footnotes

1 Bogotá is the Capital of Colombia, and the largest city in the country. It is divided in twenty localities with administrative purpose. Each locality has their own local Major and the local council. Each locality is formed of neighborhoods [51].

## Authors’ information

The first author is a Colombian physician who work as General Practitioner for 10 years in the community. She is a Professor at Pontificia Universidad Javeriana in Bogotá.
